# Supra-aortic valve tendon mimicking an acute aortic dissection: a case report

**DOI:** 10.1093/ehjcr/ytaf160

**Published:** 2025-04-08

**Authors:** Donovon Allen, Ahmed Saleh, Kais Tounsi, Alexander Yang, Min Xie

**Affiliations:** Department of Medicine, University of Alabama at Birmingham, 1808 7th Ave S, Birmingham, AL 35233, USA; Tinsley Harrison Internal Medicine Residency Training Program, University of Alabama at Birmingham, 1720 2nd Avenue South, BDB 321, Birmingham, AL 35294-0012, USA; Department of Medicine, University of Alabama at Birmingham, 1808 7th Ave S, Birmingham, AL 35233, USA; Tinsley Harrison Internal Medicine Residency Training Program, University of Alabama at Birmingham, 1720 2nd Avenue South, BDB 321, Birmingham, AL 35294-0012, USA; Department of Medicine, University of Alabama at Birmingham, 1808 7th Ave S, Birmingham, AL 35233, USA; Tinsley Harrison Internal Medicine Residency Training Program, University of Alabama at Birmingham, 1720 2nd Avenue South, BDB 321, Birmingham, AL 35294-0012, USA; Department of Medicine, University of Alabama at Birmingham, 1808 7th Ave S, Birmingham, AL 35233, USA; Department of Medicine, Division of Cardiovascular Disease, University of Alabama at Birmingham, Tinsley Harrison Tower, Suite 311, 1900 University Boulevard, Birmingham, AL 35233, USA

## Case description

**Figure 1:**
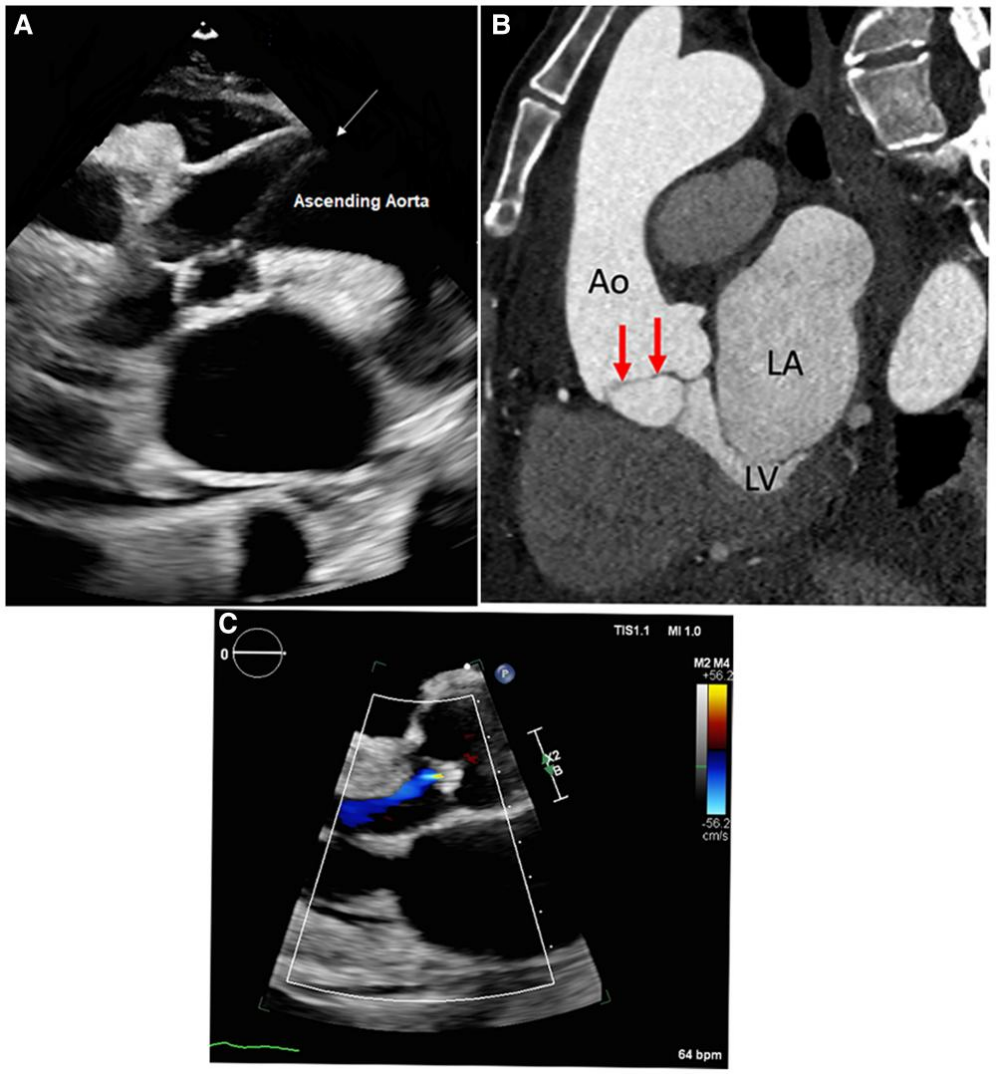
(*A*) Transthoracic echocardiogram with concern for linear echoes within the aorta suggestive of dissection (white arrow). (*B*) Sagittal oblique reformatted multiplanar image from a retrospectively gated cardiac computed tomography angiogram: There is a thin linear filling defect (inferior to Ao) along the right aortic cusp seen intermittently during the cardiac cycle without any associated aortic wall haematoma or irregularity. This corresponds to the abnormality seen on echo. (*C*) Mild aortic regurgitation demonstrated on transthoracic echocardiography. This is the most common finding associated with fibrous band. Ao, aorta; LA, left atrium; LV, left ventricle.

A 58-year-old female presented to the emergency room with a malignant gastric outlet obstruction. Her physical exam was significant for a new murmur. A transthoracic echocardiogram (TTE) was completed demonstrating linear echoes within the aorta suggestive of dissection in the aortic arch (*Figure 1A*). An emergent computed tomography angiogram (CTA) chest was obtained given the suspicion of a Type B aortic dissection but was limited due to motion artefact. A CTA-gated chest was obtained, showing a thin non-calcified linear density in the right coronary sinus seen intermittently during the cardiac cycle likely representing a supra-aortic tendon, mimicking a dissection flap (*Figure 1B*). Given that this was a benign finding, and the patient wished to pursue hospice, further interventions were not in line with her goals.

Supra-aortic tendons have been described as congenital fibrous or elastic bands typically found during autopsy or incidentally on imaging. An explanation for fibrous band formation is the incomplete remodelling of cardiac cushions during aortic valve development.^1^ They are often associated with other congenital anomalies such as bicuspid and quadricuspid valves and aortic regurgitation with and without band ruptures.^2^ In one study, a fibrous band identified on transoesophageal echocardiography had a sensitivity of 57% and 92% specificity for aortic cusp prolapse, with the most common cusp being right cusp.^3^ Our patient also exhibited mild aortic regurgitation (AR), with the fibrous band appearing to originate from the right coronary sinus (*Figure 1C*).

Current guidelines do not highlight the presence of bands as a significant concern for AR; however, they may be useful tools for identifying patients with AR and cusp prolapse.^4^ Management of incidental fibrous bands is poorly defined in the literature due to their rarity and asymptomatic nature. Suggested approaches include follow-up with TTE and surgery consultation if significant aortic valve dysfunction is present.

**Consent:** The authors confirm that written consent was obtained for publication, and all identifiable patient information has been removed from images in compliance with COPE guidelines.

**Funding:** None declared.

## Data Availability

The data underlying this article will be shared on reasonable request to the corresponding author.
